# Dietary Supplementation with Organoselenium Accelerates Recovery of Bladder Expression, but Does Not Improve Locomotor Function, following Spinal Cord Injury

**DOI:** 10.1371/journal.pone.0147716

**Published:** 2016-01-29

**Authors:** Carolyn A. Meyer, Ranjana Singh, Mackenzie T. Jones, Chen-Guang Yu, Ronan F. Power, James W. Geddes

**Affiliations:** 1 Spinal Cord and Brain Injury Research Center, College of Medicine, University of Kentucky, Lexington, Kentucky, United States of America; 2 Department of Anatomy and Neurobiology, College of Medicine, University of Kentucky, Lexington, Kentucky, United States of America; 3 Center for Animal Nutrigenomics and Applied Animal Nutrition, Alltech, Nicholasville, Kentucky, United States of America; University of Kentucky Medical Center, UNITED STATES

## Abstract

Selenium is an essential element required for activity of several antioxidant enzymes, including glutathione peroxidase. Because of the critical role of the antioxidant system in responding to traumatic events, we hypothesized that dietary selenium supplementation would enhance neuroprotection in a rodent model of spinal cord injury. Rats were maintained on either a control or selenium-enriched diet prior to, and following, injury. Dietary selenium supplementation, provided as selenized yeast added to normal rat chow, resulted in a doubling of selenium levels in the spinal cord. Dietary selenium reduced the time required for recovery of bladder function following thoracic spinal cord injury. However, this was not accompanied by improvement in locomotor function or tissue sparing.

## Introduction

Spinal cord injury (SCI) is a prevalent clinical problem, with approximately 273,000 patients living with SCI in the United States[1]. Young adults represent a particularly vulnerable population due to their active lifestyle and higher prevalence of military service. A prophylactic supplement to protect in the event of acute neurotrauma may be particularly beneficial to this high risk group.

We investigated selenium supplementation as a prophylactic treatment for reducing damage and improving functional outcomes following spinal cord injury. Selenium is essential for the formation of selenoproteins, particularly in the CNS. However, different forms of selenium are associated with varying toxicity levels[2]. Organic selenium supplements have much lower levels of toxicity and fewer of the pathogenic effects that are commonly associated with high levels of inorganic selenium[3–6]. Selenomethionine can be incorporated in place of methionine in tissues, allowing for storage of selenium in the event of a dietary deficiency.

The diets utilized in this study were formulated with selenized yeast, Sel-Plex^®^, to provide an organic form of selenium supplementation. The selenium is present in the form of two amino acids, selenomethionine and selenocysteine, as well as a variety of other seleno-compounds[7]. Selenized yeast has significantly lower toxicity than sodium selenite[8]. High levels of inorganic selenium in the form of selenite can have detrimental neurological effects [9], however these results have not been demonstrated with selenized yeast. In contrast, several studies have shown beneficial effects of selenium in the progression of neurodegenerative diseases [10–12].

The brain and spinal cord prioritize the retention of selenium when dietary levels are deficient[13], utilizing the selenium transporter, selenoprotein P (SelP), to supply physiological selenium to the CNS[14, 15]. Selenoproteins are well characterized for their role in redox regulation[16–18] and anti-inflammatory pathways[10, 19], both of which are implicated in the secondary injury cascade following SCI [20–23]. In particular, several antioxidant enzymes, including glutathione peroxidases and thioredoxin reductases, are selenoproteins, which require selenocysteine for protein production. Reactive oxygen species initiate lipid hydrolysis, breakdown of cellular membranes, mitochondrial dysfunction, and thus perpetuate the damaging secondary injury cascade [24]. Increasing levels of selenium available in tissues prior to injury may prepare the CNS to quickly synthesize selenoproteins to mitigate this series of damaging events.

The objective of this study was to examine the effect of supplementation with selenized yeast on functional and pathological endpoints following SCI. Dietary selenium supplementation increased selenium tissue storage in the CNS, with no aberrant weight gain or other adverse physiological changes between treatment groups. This increase in selenium levels did not translate to a visible improvement in locomotion or lesion volume in the injured tissue, but resulted in more rapid recovery of autonomous bladder expression.

## Materials and Methods

### Animal care and diet

Female Sprague-Dawley rats were obtained from Harlan at weaning and placed immediately on either a control diet or on a selenium enriched diet (Se-yeast, Sel-Plex, Alltech, Nicholasville, KY). The selenium enriched diet also used a standard rat chow as the diet base and incorporated a selenized yeast preparation (1.3ppm total selenium, approximately 19.5μg per day) in which the yeast is grown in the presence of selenium. The control diet of normal rat chow contained standard dietary levels of selenium (0.3ppm selenium, approximately 4.5μg per day) and a yeast additive to account for the presence of selenized yeast in the selenium enriched diet. The LD_50_ of acute administration of Sel-Plex is ≥2500mg/kg in rats and the overall no observed adverse effect level (NOAEL) is 30mg/kg/day whereas similar levels of inorganic sodium selenite resulted in mortality and clinical pathologies. Animals were fed their respective diets (n = 20 per diet) *ad libitum* for 16 weeks. In a separate study, animals received the diets for 4 weeks, 8 weeks or 16 weeks followed by evaluation of Se levels in the spinal cord. Throughout the trials, animals were weighed weekly, and immediately prior to receiving the injury, to monitor health statuses of the rats as well as to check for any significant differences in body weight of the animals at the time of injury. All animal procedures were approved by the University of Kentucky Institute of Animal Care and Use Committee (IACUC).

### Spinal cord injury and post-surgical care

Following 4 months of dietary supplementation, rats were subjected to a moderate contusive SCI (150kdyn). Contusive SCI surgeries were performed as previously described[25]. In brief, rats were anesthetized with an intraperitoneal injection of ketamine (80mg/kg) and xylazine (10mg/kg). The spinal cord was exposed via a T10 laminectomy. The rats received a moderate (150kdyn) contusive thoracic SCI using the Infinite Horizons SCI injury device (Precision Systems and Instrumentation). Sham animals were handled identically, including the laminectomy procedure, but did not receive the thoracic SCI. Following SCI (n = 12) or sham surgery (n = 8), the musculature and skin were closed with sutures and wound clips, respectively. As part of routine post-surgical care, bladders were manually expressed twice daily using the Credé maneuver[26] until the animal recovered autonomous bladder control. Bladder function following injury was recorded as either non-functional (full bladder) or functional (empty to half-full bladder) prior to manual voiding [27]. The number of days until each animal exhibited autonomous bladder functional recovery was recorded. The rats also received injections of Buprenorphine (0.05 mg/kg) twice daily to manage pain and Baytril (5-10mg/kg) twice daily for three days post injury to prevent development of post-surgical infection. Animals were maintained on their respective diets until the time of euthanasia.

### Spinal cord and cortical selenium levels

A separate cohort of animals was also maintained on the two different diets for 1 to 4 months under the conditions as described above. For the SCI studies, animals were fed *ad libitum* for 4 months (n = 5 per dietary group). Immediately following the feeding regimen animals received the moderate contusive spinal cord injury described above. 24 hours post injury, fresh tissue from spinal cord sections both rostral and caudal to the injury site and cortical samples were removed and flash frozen in liquid nitrogen. Selenium levels were then analyzed by liquid chromatography-mass spectroscopy (LC-MS). Values rostral and caudal to the injury site were averaged for each animal. One value was removed from spinal cord dataset due to being an extreme outlier (greater than 3 standard deviations from the mean).

### Behavioral assessment

At three days following SCI, two investigators blinded to treatment groups assessed open field locomotor functional recovery using the Basso, Beattie, and Bresnahan (BBB) locomotor rating scale[28]. Locomotor testing was then repeated once weekly for 6 weeks following injury. At 3 days following injury there is a substantial injury effect. With a moderate injury animals lose motor control of their hind limbs. Over 6 weeks, rats typically demonstrate a gradual improvement in locomotion that typically plateaus around a BBB score of 13, indicating that animals have frequent to consistent weight support in plantar stepping and frequent coordination. When discrepancy occurred between observers, the lower score was assigned to the animal’s performance.

### Tissue histology

Following the final behavioral testing, animals were given an intraperitoneal injection of a fatal overdose of sodium pentobarbital and then perfused with ice-cold phosphate buffered saline (PBS), followed by fixation with a 4% paraformaldehyde/PBS solution. Spinal cord tissues were kept at 4°C in a 4% paraformaldehyde solution for 4 hours and then transferred to a 30% sucrose solution overnight at 4°C for cryopreservation. The fixed tissues were transferred into OCT medium for cryosectioning at -25°C. Tissue histology was evaluated in a subset of animals randomly selected from each group (n = 8 for each injury group, n = 5–7 for sham injured groups). Transverse spinal cord sections (20μm thick) were collected every 100μm and mounted onto microscope slides (Fisher Superfrost). Sections were stained utilizing eriochrome cyanine RC and cresyl violet [29, 30]. Areas of spared and lesioned tissue were measured for sections extending 0.7mm on either side of the injury epicenter using Scion Imaging analysis software (Scion Corporation, Frederick, MD). Utilizing the Cavalieri method[31], the total volume of tissue in the lesion or in spared tissue was calculated. The investigator was blinded to treatment groups until all parameters had been measured and calculated.

### Statistical Analysis

Data represented in each figure are shown as mean±standard deviation. Using GraphPad Prism 6.0, differences in weekly weight gain were evaluated by repeated measures, one-way ANOVA encompassing the 16 week feeding period. Additionally, Student’s t-test (significance at p<0.05) was used to compare final weights, and also time to autonomous bladder function in the SCI groups. Differences in behavioral assessment (BBB Scores) between treatment groups were assessed by mixed factorial ANOVA with significance assigned at *p*<0.05 [32]. As the two sham groups had the same response on each of the days, the ANOVA was conducted on the SCI+SelPlex and SCI-control diet groups. Tissue selenium content was compared using Student’s t-test (*p*<0.05) between control and selenium-enriched diets. A two-way ANOVA evaluating the six week scores of locomotor function and time to recovery of autonomous bladder function was also performed.

## Results

### Animal weights

**Fig 1:**
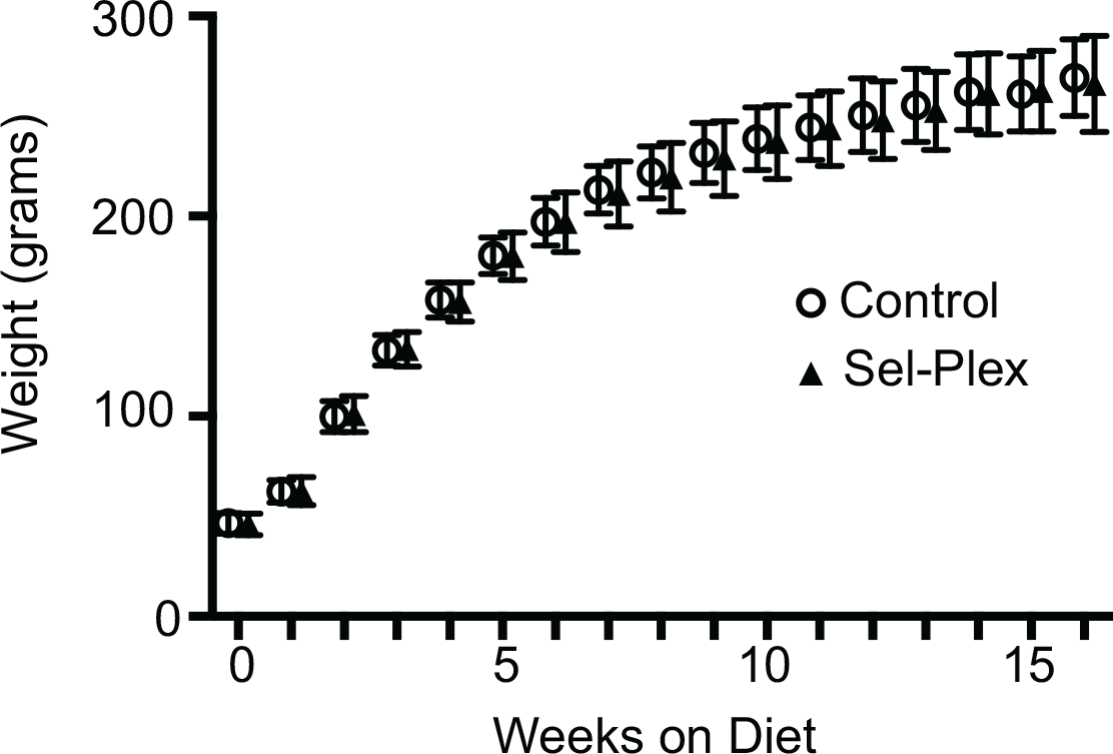
Dietary selenium enrichment does not affect overall weight gain. Rats were weighed weekly. No differences in body weight were seen between selenium enriched rats and rats maintained on control diet (n = 25).

To ascertain the effect of the selenium-enriched and control diets on body weight throughout the course of the study, animals were weighed weekly as described above (n = 25 for each diet). Weight gain showed no significant differences between rats maintained on the control (+yeast) rat chow and the yeast-selenium enriched diet (Fig 1).

### Selenium levels

**Fig 2:**
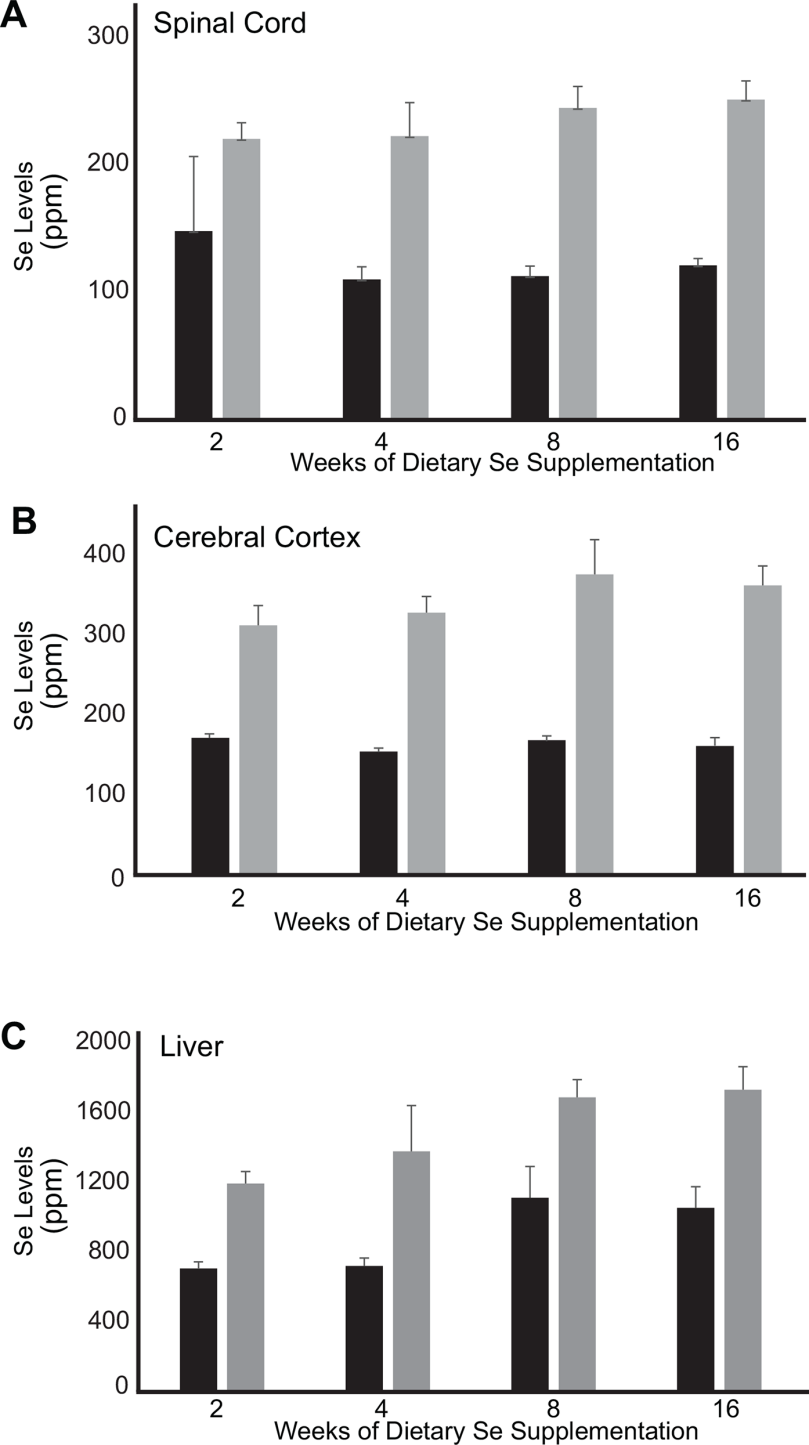
Dietary selenium enrichment increases selenium tissue storage. CNS and liver selenium levels were analyzed by LC-MS. The results indicate that supplemented selenium crosses the blood-brain barrier and is available for incorporation into spinal cord and cortical tissue. Values are mean±SD, n = 5/group.

Data from liquid chromatography mass spectroscopy (LC-MS) showed that dietary enrichment with selenium increased tissue storage of selenium. In the spinal cord, cortex, and liver, selenium levels increased by approximately 2-fold between the control diet and selenium enriched diet (Fig 2). The magnitude of increased tissue storage was similar for animals on the selenium enriched diet for one, two and four months.

### Bladder function

**Fig 3:**
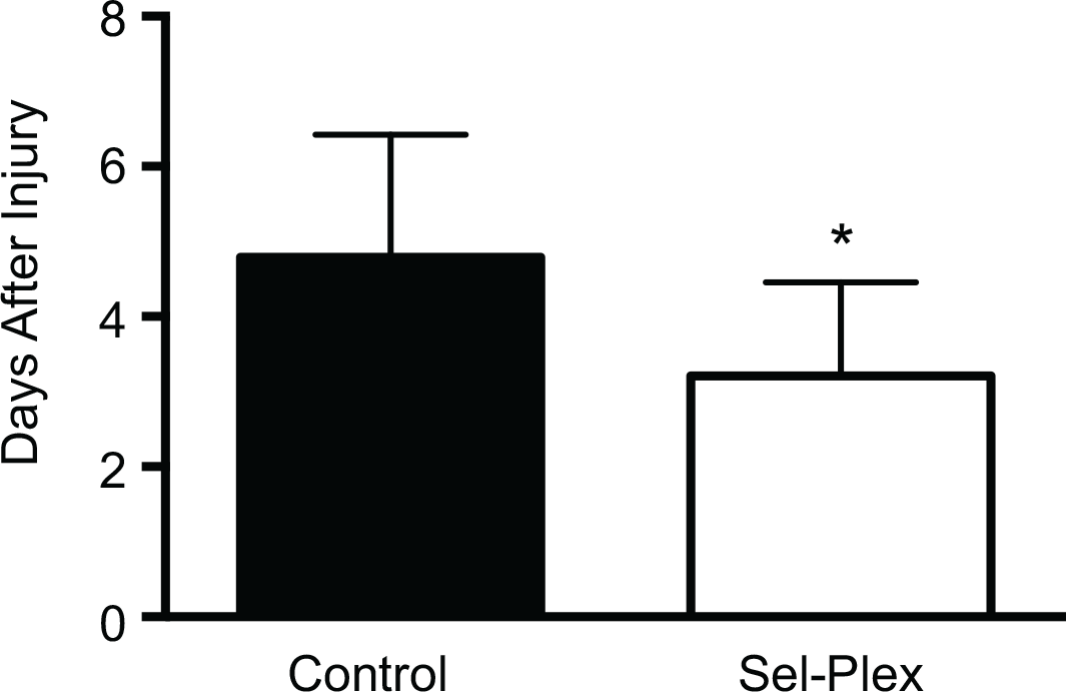
Dietary selenium supplementation improves the time to recovery of bladder function. Rats receiving selenium supplementation recovered bladder function in an average of 3 days±0.4 as compared to rats on a control diet, which recovered bladder function in 5 days±0.5. Recovery of bladder expression is an additional functional marker for improvement. Values are mean±SD, p<0.05.

Animals (n = 12 per diet) maintained on selenium enriched diets regained bladder function by 3±0.4 days after injury as compared to rats on a control diet, at 5±0.5 days (*p*<0.05) (Fig 3). Animals that received sham surgeries did not lose autonomous bladder control.

### Locomotor functional recovery

**Fig 4:**
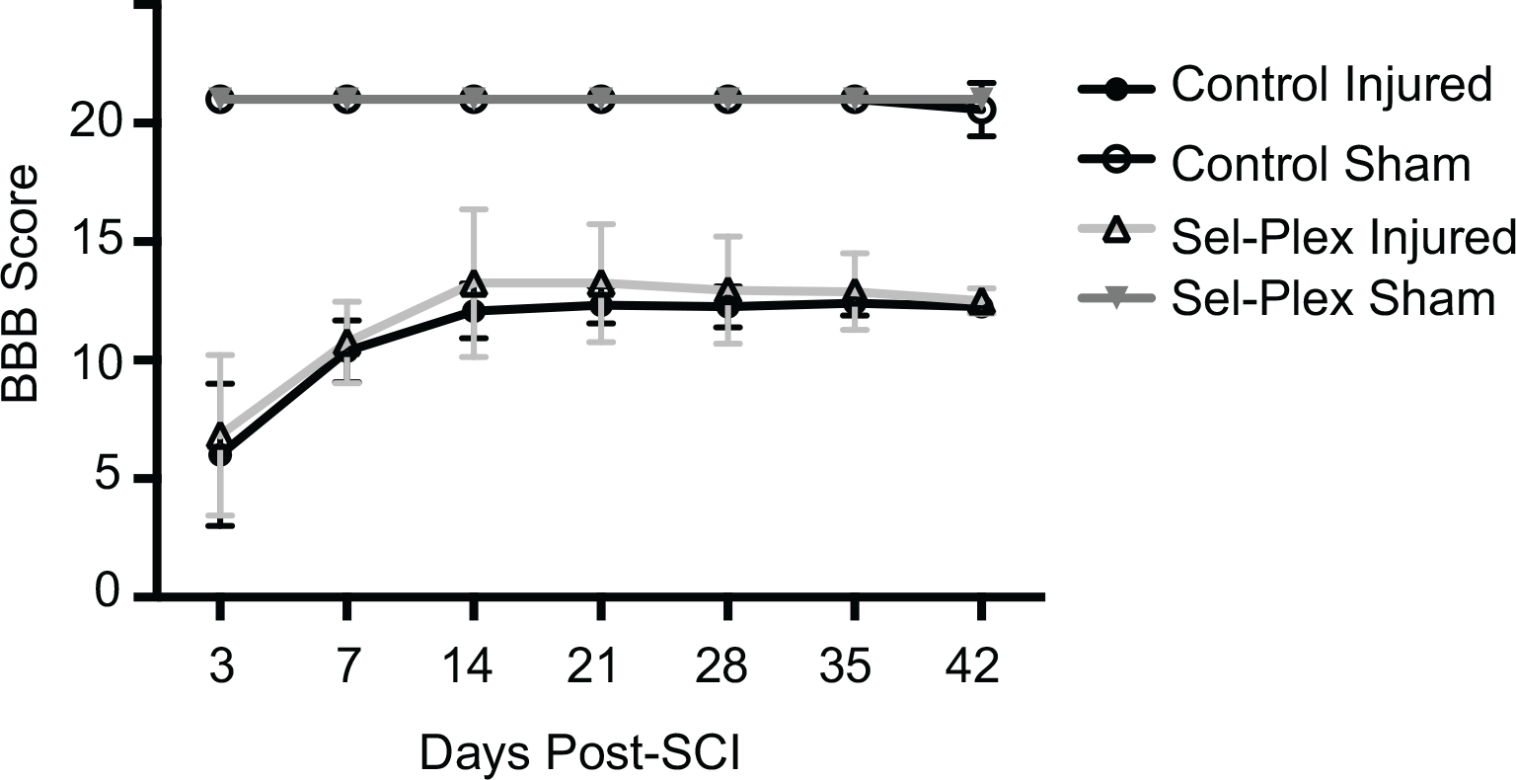
Dietary selenium supplementation does not improve locomotor functional recovery. Recovery was evaluated weekly after injury using the BBB scale. No difference in improvement was seen between the two diets in the injured rats. Sham animals showed no change in performance after laminectomy. Values are mean±SD (n = 12 injured, n = 8 sham).

Injured animals in both groups had a complete loss of hindlimb locomotor function immediately following surgery, confirming that the contusion injury was effective. Three days after injury, all rats exhibited slight motor recovery, as evident from the BBB scores, which reflect slight movement of two joints and extensive movement of the third joint. Over the course of the 6 week behavioral testing, all injured animals showed a steady improvement in locomotor function, plateauing around the second week with overall function that included consistent stepping, occasional to frequent coordination, and occasional correct paw placement. In the injured animals, there was no significant difference in locomotor functional recovery, over time or during the final behavioral testing, between those fed the control diet and animals fed the selenium-enriched diet (Fig 4). Sham animals in both dietary groups showed no loss of locomotor function following sham surgery. We also compared the six week BBB scores with time to recovery of bladder function using a two-way ANOVA. The results were significant (p<0.05) for interaction, diet, and functional marker.

### Tissue lesion volume

**Fig 5:**
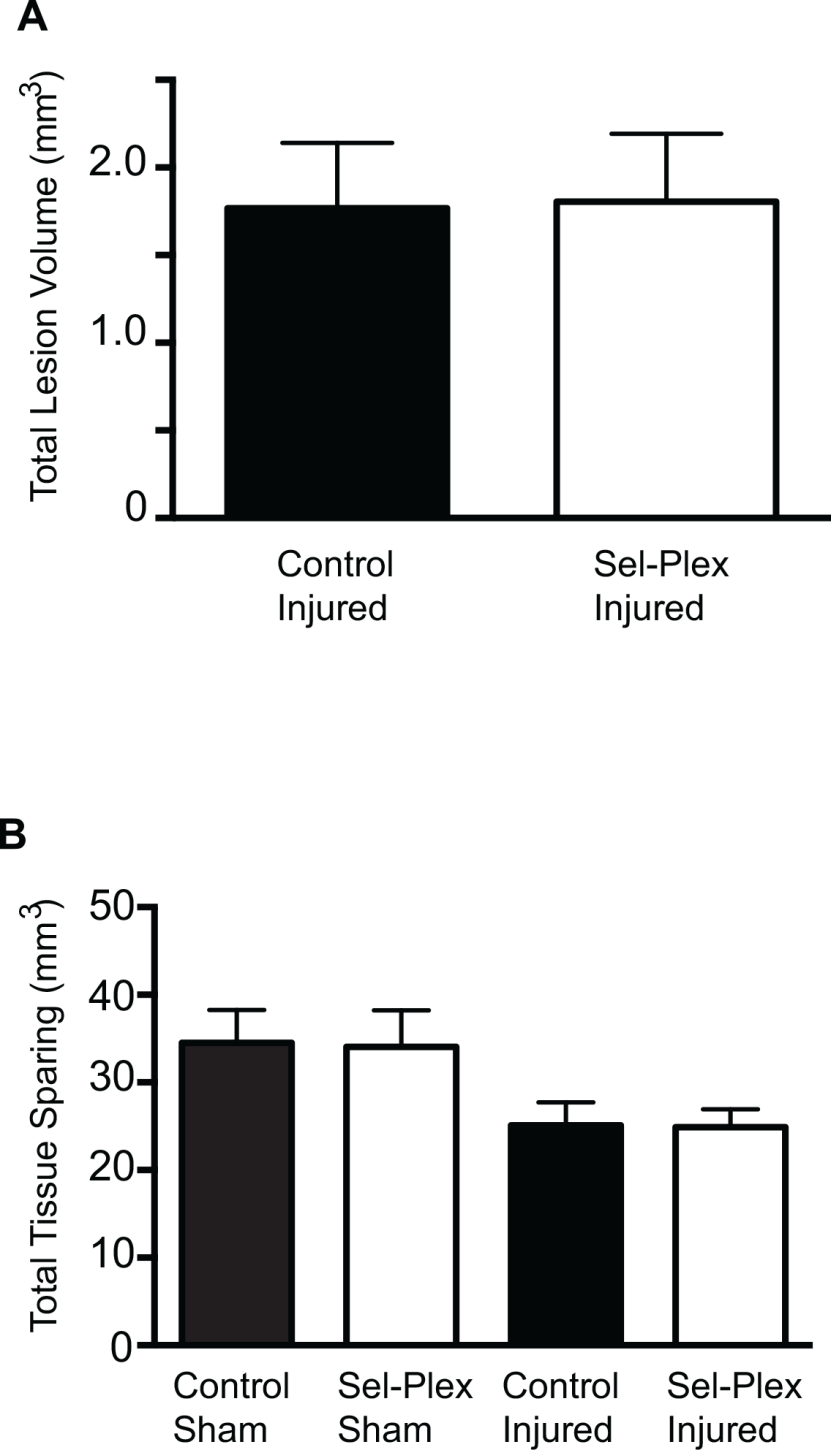
Dietary selenium supplementation does not reduce lesion volume following SCI. Spinal cords sections (20μm) were stained for cell bodies and myelin. Total lesion volume (A) and total tissue sparing (B) revealed a significant injury effect (p<0.01) between sham and injured. No significant difference in lesion volume or tissue sparing was seen between the control injured and the selenium injured group. Additionally, there were no significant differences in grey matter sparing and white matter sparing (n = 12 injured).

Staining for myelin and neuronal cell bodies showed no significant difference between the two injured groups in total lesion volume, or in the total amount of gray matter and white matter sparing after injury (Fig 5). Sham injured animals showed no tissue lesion.

## Discussion

The CNS maintains a high priority for selenium storage in deficient conditions, suggesting a critical role of selenium in CNS tissues [13, 33–35]. Dietary selenium supplementation has been examined previously for metastatic brain tumors[36], Alzheimer’s Disease[37], ischemia[38], and Parkinson’s Disease[39] and TBI [40]. One previous study examined the effects of selenium on SCI [41], although the experimental conditions differed markedly. Yeo and colleagues injected sodium selenite (10-50ng/kg) mixed with matrigel directly into the lesion site immediately following a dorsal hemisection injury. This study observed a reduction in apoptotic cell death, decreased glial fibrillary acidic protein (GFAP) positive cells, and a dramatic improvement in locomotor function in selenium treated rats. Selenium supplementation has also been recently evaluated in a model of ischemic/reperfusion injury to the rat spinal cord following blockage of the abdominal aorta [42]. The rats received feed containing 5 mg/L selenium for two weeks prior to experimentation, tissue levels were not evaluated. Rats were euthanized 7 days following a 30 minute blockade of the abdominal aorta. Selenium supplementation increased cell survival, decreased necrosis and edema, an increased expression of ciliary neurotrophic factor (CNTF) and it’s receptor α (CNTF-Rα) [42].

Pre-injury dietary supplementation in this study resulted in increased CNS tissue levels of selenium, providing increased bioavailability of seleno-amino acids for the production of selenoproteins. However, the increased selenium levels did not result in improved tissue sparing. In behavioral tasks, the rats receiving a selenium-enriched diet did not exhibit significant improved locomotor function post-injury as compared to animals whose diet consisted of normal rat chow.

Rats in the selenium enriched diet group were able to regain autonomous control of bladder expression following spinal cord contusion injury more quickly than rats maintained on the control diet. Initiation of autonomous micturition has been examined as a marker for recovery of sensory-motor function in previous spinal cord contusion studies [43, 44].

Neurological control of bladder function is controlled through splanchnic parasympathetic nerves (located in the sacral spinal cord S2-S4), pudendal nerves (also in the sacral spinal cord S2-S4), and thoracic sympathetic nerves (cell bodies originating at T10-L2 spinal cord levels). Sympathetic innervation of the bladder plays a crucial role in closing the internal urethral sphincter and of blood vessels in the detrusor muscle of the bladder. Although histological examination did not show an overall improvement in total lesion volume, it is possible that sympathetic neurons present at the site of injury (thoracic level T10) were protected with selenium treatment.

Although histological examination did not show an overall improvement in total lesion volume, it is possible that sympathetic neurons present at the site of injury (thoracic level T10) were protected with selenium treatment. Ferrero and colleagues noted that the closer a lesion was to T10, the slower the bladder recovery time [45]. They also commented that because of the involvement of this region in sympathetic and sensory innervation of the ureter and kidney, lesions in this area should be avoided for SCI investigations of micturition pathways.

In rat contusion (incomplete injury) models of SCI, rats spontaneously recover voluntary bladder control in the days to weeks following injury. Human patients with incomplete SCI do not exhibit recovery of voluntary bladder control. Disruptions in bladder and bowel function are very important clinical pathologies to SCI patients, however, disconnect between clinical outcomes and those found in experimental models may limit the translation of bladder functional recovery in preclinical models to patient application.

Overall, the results do not support our hypothesis that selenium supplementation would result in improved outcomes following SCI. However, findings indicating bladder functional recovery without associated improvement in locomotor behavioral tasks suggest that these improvements in bladder function may be important markers for functional recovery[46, 47].

Current studies are examining effects of selenium-enriched and selenium-deficient diets on levels of selenoproteins and related enzyme activities in both naïve and traumatic brain injured animals. The antioxidant natures of many of the selenoproteins in the CNS, along with the results from this study suggest, that while selenium’s effects may be modest in this SCI model, examining various levels of selenium to attenuate neurodegenerative pathways warrants further research.
